# The state of human papillomavirus research in Africa

**DOI:** 10.1002/puh2.72

**Published:** 2023-03-03

**Authors:** Kehinde Kazeem Kanmodi, Eyinade Adeduntan Egbedina, Jimoh Amzat, Kafayat Aminu, Lawrence Achilles Nnyanzi

**Affiliations:** ^1^ School of Health and Life Sciences Teesside University Middlesbrough UK; ^2^ Cephas Health Research Initiative Inc Ibadan Nigeria; ^3^ End Cervical Cancer Nigeria Initiative Inc Birnin Kebbi Nigeria; ^4^ Faculty of Dentistry University of Puthisastra Phnom Penh Cambodia; ^5^ Department of Sociology Usmanu Danfodiyo University Sokoto Nigeria; ^6^ Department of Sociology University of Johannesburg Johannesburg South Africa; ^7^ Centre for Child and Adolescent Mental Health University College Hospital Ibadan Nigeria; ^8^ School of Public Health King Ceasor University Kampala Uganda

**Keywords:** Africa, bibliometric review, human papillomavirus, policy, research, strategy

## Abstract

**Background:**

Human papillomavirus (HPV) research scholarship evaluation is pivotal to the strategic planning, implementation and sustainability of HPV prevention and control programmes in Africa. Hence, this study evaluated HPV research scholarship in Africa.

**Methods:**

This review—a bibliometric analysis—investigated the trends, patterns, dynamics and funding of HPV‐related literature production in Africa with a focus on the inequalities existing across thematic and subject areas, researchers, institutions and countries/territories/dependencies. The study data were obtained from SCOPUS database and analysed using the Microsoft Excel 2021 software.

**Result:**

From 1974 (inception year) till 16 July 2022, a total of 2587 SCOPUS‐indexed literature on HPV were produced by African authors, with an average production rate of 50.5 publications per year (1974–2021). A few publications (1.2%) were in the Social Sciences. Most (95.1%) publications were in English, only a few (3.9%) were in French, whereas none was in Arabic, Portuguese, Spanish, Swahili or any other official language of the African Union. South Africa, Nigeria and Egypt were the three most prolific African countries. The 10 most prolific researchers were affiliated to public institutions in South Africa, Tanzania and Botswana. The top 10 funding sponsors were public institutions in the United States, the United Kingdom, Belgium and South Africa. Moreover, researchers and institutions affiliated to South Africa, Nigeria, Kenya and Uganda were the predominant beneficiaries. Only two indigenous journals made the list of top 10 journals publishing HPV research outputs from Africa.

**Conclusion:**

Scholarly HPV research productivity in Africa is very low and worsened by multiple inequality gaps. There is an urgent need for equitably strengthening HPV research capacity in Africa.

## INTRODUCTION

The human papillomavirus (HPV) is a human pathogenic virus with a double‐stranded deoxyribonucleic acid (DNA) [1]. Over 100 known types of HPVs, and they are grouped into non‐carcinogenic and carcinogenic types [2]. The common non‐carcinogenic types include HPV types 6, 11, 40, 42, 43, 44 and 54; these HPV types atypically cause cancers [2, 3], and typical examples of the diseases caused by them are papilloma, verruca, condyloma and focal epithelial hyperplasia (Heck's disease) [4]. On the other hand, the common carcinogenic types include HPV types 16, 18, 31, 33, 35, 39 and 45 [2, 3], and they are known for causing oropharyngeal, anal and genital cancers [1, 2, 3, 4, 5].

HPV can be transmitted congenitally (at childbirth), and through sexual intercourse (fellatio, cunnilingus, anilingus and vaginal/anal penetrative sex) and direct skin‐to‐skin contact [1, 4, 6–8]. This shows that HPV is a highly transmissible virus.

Research evidence has shown that most human HPV infections are asymptomatic in both genders [9]. However, HPV infection still accounts for the majority (or a significant percentage) of cervical (∼100%), anal (∼90%), vaginal (∼70%), penile (∼50%), vulvar (∼40%) and oropharyngeal (∼13%–72%) cancers [10, 11, 12, 13, 14, 15, 16].

African countries have the greatest burdens of HPV‐induced diseases [3, 17, 18]. For example, the prevalence rate of HPV infection among African women is 21.1%; this rate is higher than that recorded in other regions except for Oceania, which has a prevalence rate of 21.8% [18]. Overall, this demonstrates that the HPV situation in Africa is an issue of a public health emergency. However, many factors, including low awareness and knowledge rates on HPV, low HPV vaccination rates, weak public health policies and systems, low political will, poverty and poor collaborative research culture, are responsible for this HPV‐associated calamity in Africa [19, 20, 21, 22, 23].

Overall, scholarly research is pivotal in the strategic planning, implementation and sustainability of HPV prevention and control programmes in Africa [20, 21, 24, 25]. Several studies, adopting different research designs and ranging from KAP (knowledge, attitude and practice) studies to molecular‐based studies, have been conducted in Africa on HPV. However, no known study has evaluated the state of HPV research scholarship in Africa.

A bibliometric analysis approach can be used to evaluate the state of HPV research scholarship in Africa [26, 27, 28, 29]. Through this approach, the scope, issues, challenges and prospects of this scholarly area can be well elucidated, and this will aid a profound understanding of the research landscape in Africa [26, 27, 28, 29]. The study outcomes will form a base for further developing scholarly HPV research capacity on the African continent [26, 27, 28, 29].

## METHODS

This study was a bibliometric analysis of HPV research in Africa and was conducted based on the approach recommended by Donthu et al. [28]. The SCOPUS database was the sole database used for this study; this was because the database is the oldest, most comprehensive, most superior and most widely used database for bibliometric analyses [26, 27, 28, 29]. Moreover, only one database was used for this study due to the plan to conduct science mapping of bibliometric data—the use of multiple databases significantly limits the opportunity for science mapping [26, 27, 28, 29, 30, 31].

On 16 July 2022, a SCOPUS‐based search was conducted to scoop out publications on HPV, (co‐)authored by African researchers using the search string depicted in Box S1. The bibliometric data of the publications obtained were extracted from SCOPUS in a comma‐separated value (.csv) format.

The obtained bibliometric data include bibliographic data (territorial and institutional affiliations, and languages); citation information (source, type, title, author, volume, issue, pagination and citation counts), funding sponsor(s), *h*‐index, CiteScore 2021 (as defined by SCOPUS) and subject area(s) of each publication [28].

The collected data were analysed using Microsoft Excel version 2021 and VOSviewer 1.6.18 software. The performance analysis of the bibliometric metrics was done using Microsoft Excel, whereas VOSviewer was used for science mapping.

## RESULTS

**FIGURE 1:**
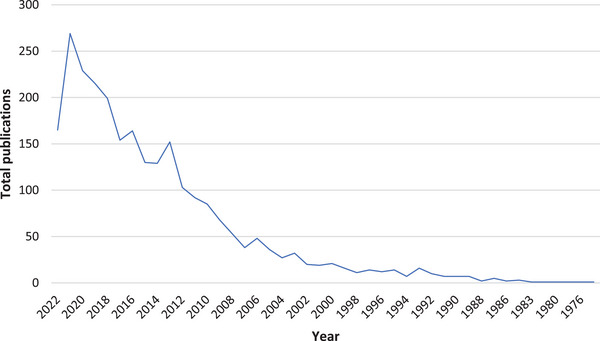
Trend analysis of annual publications on human papillomavirus (HPV) in Africa, 1974–2022.

A total of 2587 publications were retrieved—all were published between 1974 and 16 July 2022. The trend analysis of the annual production rate of these publications shows a progressive increase (Figure 1).

**FIGURE 2:**
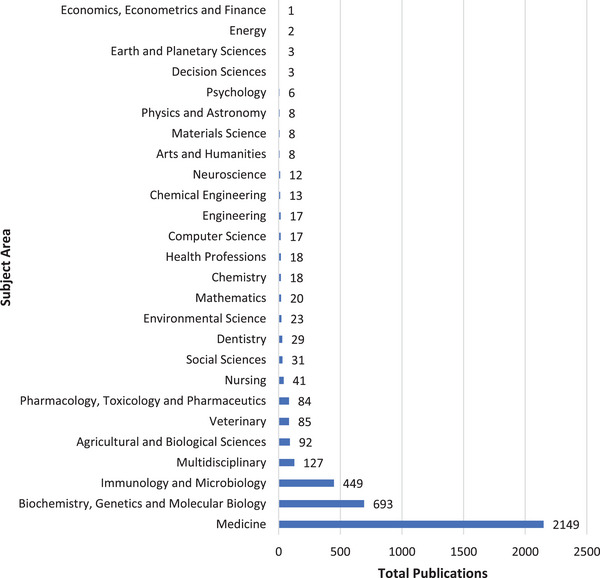
Distributions, by subject area, of publications on human papillomavirus (HPV) in Africa, 1974–2022.

The majority (83.1%) of these publications were classified under the Medicine subject area; only a few were classified under Social Sciences (1.2%), Dentistry (1.1%), Health Professions (0.7%) and Psychology (0.2%) subject areas (Figure 2).

**FIGURE 3:**
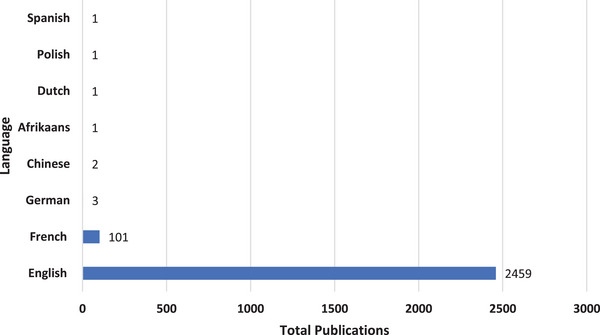
Distributions, by publication language, of publications on human papillomavirus (HPV) in Africa, 1974–2022.

All these publications were published in eight languages, of which only one language (African) was an African language. The top three languages of publication were English (95.1%), French (3.9%) and German (0.1%) (Figure 3).

The majority (98.5%) of these publications were published in journals (Table 1). However, the total citations (TC), average citations per publication (ACP) and *h*‐index of the obtained journal publications could not be determined because SCOPUS does not provide data on the immediate cumulative TC and *h*‐index data for a collection of publications over 2000 at a go. However, the data on the TC, *h*‐index and ACP of other publication types were obtained and analysed. Among other publication types, book series were found to have the highest TC (178), ACP (19.8) and *h*‐index (4) (Table 1).

**TABLE 1:** Distributions, by publication type, of publications on human papillomavirus (HPV) in Africa

Source type (*n* = 2587)	Total publications (%)	Total citations	Average citation per publication	*h*‐Index
Journal	2549 (98.5)	DTL^a^	DTL^a^	DTL^a^
Book	19 (0.7)	20	1.1	3
Book series	9 (0.3)	178	19.8	4
Conference proceeding	7 (0.3)	10	1.4	2
Trade journal	1 (<0.1)	1	1	1
Undefined	2 (<0.1)	11	5.5	1

*Note*: *n*, total number of HPV publications in Africa.

Abbreviation: DTL, data too large.

^a^ SCOPUS does not generate these data for a group of publications above 2000.

Among those journal publications on HPV, articles were found to constitute the majority (82.1%) of them (Table 2). However, the TC, ACP and *h*‐index of these articles could not be determined because they were more than 2000 in quantity. Amongst other journal publication types, reviews were found to have the highest TC (8536), ACP (28.4) and *h*‐index (52) (Table 2).

**TABLE 2:** Distributions, by paper type, of journal publications on human papillomavirus (HPV) in Africa

Rank^a^	Publication type (*n* = 2549)	Total publications (%)	Total citations	Average citation per publication	*h*‐Index
First	Article	2093 (82.1)	DTL^b^	DTL^b^	DTL^b^
Second	Review	301 (11.8)	8536	28.4	52
Third	Letter	56 (2.2)	259	4.6	10
Fourth	Note	48 (1.9)	295	6.1	9
Fifth	Conference paper	36 (1.4)	482	13.4	13
Sixth	Book chapter	22 (0.9)	26	1.2	4
Seventh	Editorial	17 (0.7)	112	6.5	7
Eighth	Erratum	10 (0.4)	1	10	1
Ninth	Short survey	4 (0.2)	196	49	3

*Note*: *n*, total number of journal publications on HPV in Africa.

Abbreviation: DTL, data too large.

^a^ Ranking was based on TP.

^b^ SCOPUS does not generate these data for a group of publications above 2000.

The distributions, by the quantity of HPV publications, of the African and foreign countries sourcing publications on HPV in Africa are depicted in Figure 3, Tables 3 and 4. Only very few African countries sourced 50 publications each. The countries in the Global North were the most prolific collaborating foreign countries in HPV research in Africa (Figure 3).

**TABLE 3:** Top three most productive collaborating countries, institutions and authors

Top‐three non‐African collaborating countries							
Country (*n* = 2587)	Rank^a^	Continent	Official language	Total publications (%)	Total citations	Average citation per publication	*h*‐Index
United States	First	North America	English	821 (31.7)	29,497	35.9	80
United Kingdom	Second	Europe	English	275 (10.6)	9704	35.3	48
France	Third	Europe	French	264 (10.2)	14,573	55.2	56

Top three non‐African Collaborating Institutions							
Institution	Rank^b^	Ownership	Location	Total publications (%)	Total citations	Average citation per publication	*h*‐Index
International Agency for Research on Cancer	First	Public	France	124 (4.8)	8854	71.4	45
London School of Hygiene and Tropical Medicine	Second	Public	UK	107 (4.1)	3014	28.2	29
University of North Carolina at Chapel Hill	Third	Public	USA	93 (3.6)	3231	34.7	27

*Note*: *n*, total number of HPV publications in Africa.

Abbreviation: HAWAI, History of Affiliation with African Institution(s).

^a^ Information was obtained from the SCOPUS profile page of the author.

^b^ Ranking was based on total publications.

**TABLE 4:** Top‐three most productive African countries, dependencies or territories (CDT)

Top‐three CDT (*n* = 2587)	Rank^a^	Official national language	Total publications (%)	Total citations	Average citation per publication	*h*‐Index
**Top three African CDT**						
South Africa	First	English	819 (31.7)	20,742	25.3	70
Nigeria	Second	English	246 (9.5)	8032	32.6	35
Egypt	Third	Arabic	239 (9.2)	3010	12.6	28
**Top three CDT in North Africa**						
Egypt	First	Arabic	239 (9.2)	3010	12.6	28
Morocco	Second	Arabic, Amazigh	126 (4.9)	1856	14.7	21
Tunisia	Third	Arabic	90 (3.5)	839	9.3	17
**Top three CDT in West Africa**						
Nigeria	First	English	246 (9.5)	8032	32.7	35
Ghana	Second	English	71 (2.7)	971	13.7	13
Senegal	Third	French	45 (1.7)	1020	22.7	15
**Top three CDT in Central Africa**						
Cameroon	First	French	75 (2.9)	1006	13.4	19
Congo	Second	French	33 (1.3)	790	23.9	13
Gabon	Third	French	29 (1.1)	292	10.1	10
**Top three CDT in East Africa**						
Kenya	First	English	195 (7.5)	3388	17.4	32
Uganda	Second	English	171 (6.6)	6382	37.3	39
Tanzania	Third	English	125 (4.8)	2670	21.4	32
**Top three CDT in Southern Africa**						
South Africa	First	English	819 (31.7)	20,742	25.3	70
Zimbabwe	Second	English	75 (2.9)	565	7.5	12
Botswana	Third	English	50 (1.9)	1729	34.6	24

*Note*: *n*, total number of HPV publications in Africa.

Abbreviation: CDT, country, dependency or territory.

^a^ Ranking was based on TP.

The top three foreign countries were the United States (TP = 821; TC = 29,497; ACP = 35.9; *h*‐index = 80), United Kingdom (TP = 275; TC = 9704; ACP = 35.3; *h*‐index 48) and France (TP = 264; TC = 14573; ACP = 55.2; *h*‐index = 56) (Table 3).

The top three with the highest number of publications were South Africa (TP = 819; TC = 20,742; ACP = 25.3 and *h*‐index = 70), Nigeria (TP = 246; TC = 8032; ACP = 32.6; *h*‐index = 35) and Egypt (TP = 239; TC = 3010; ACP = 12.6; *h*‐index = 28) (Table 4). Anglophone countries were the top two prolific countries in each of the African sub‐regions except for North Africa and Central Africa (Table 4).

**FIGURE 4:**
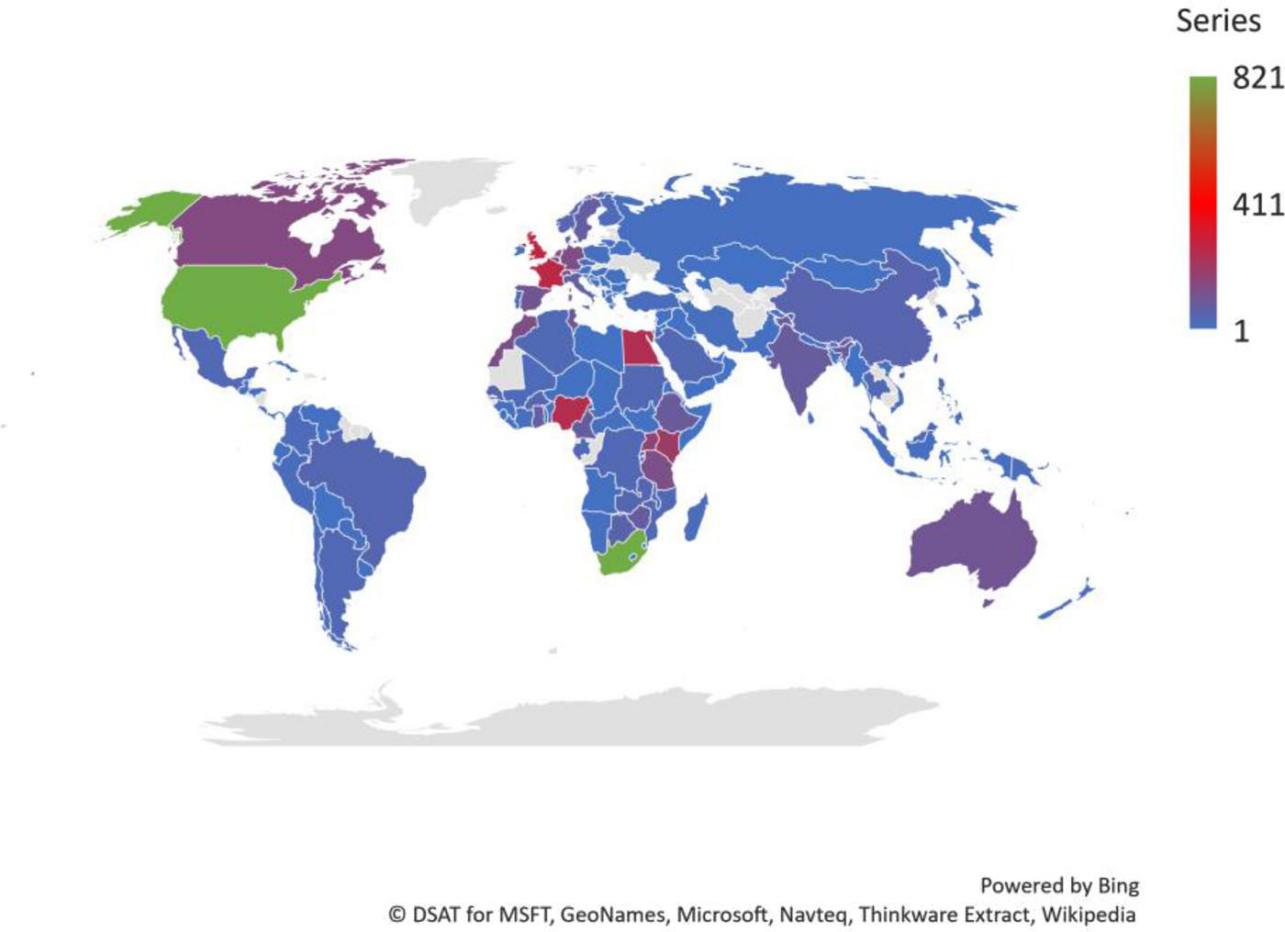
Distribution, by the quantity of human papillomavirus (HPV) publications, of African and foreign countries sourcing publications on HPV in Africa, 1974–2022.

The most prolific African and foreign institutions sourcing HPV publications in Africa are depicted in Figure 4, Tables 3 and 5. The top three foreign institutions were public‐owned and based in the Global North: France, the UK and the United States. The International Agency for Research on Cancer was the most prolific of them all (TP = 124; TC = 8854; ACP = 71.4; *h*‐index = 45) (Table 3). The top‐three most prolific African institutions were public‐owned and based in South Africa (Table S1). The University of Cape Town was the most prolific of them all (TP = 335; TC = 11,355; ACP = 33.9; *h*‐index = 55). Furthermore, in all the five African sub‐regions, only public‐owned institutions made the top‐three most prolific institutions (Table S1).

**TABLE 5:** The top 10 sponsors of human papillomavirus (HPV) research projects in Africa

Rank^a^	Sponsor	Country HQ	Ownership	TSP (%TPA)	Total citations	Average citation per publication	*h*‐Index	TPAHQCS (%TSP)	Country 1 (*N*, %TSP)	Country 2 (*N*, %TSP)	Country 3 (*N*, %TSP)
First	National Institutes of Health	USA	Public	266 (10.3)	7860	29.5	44	245 (92.1)	South Africa (66%, 24.8%)	Kenya (49%, 18.4%)	Nigeria (39%, 14.7%)
Second	National Cancer Institute	USA	Public	207 (8.0)	7900	38.2	46	199 (92.1)	Kenya (47%, 22.7%)	South Africa (41%, 19.8%)	Nigeria (27%, 13.0%)
Third	National Institute of Allergy and Infectious Diseases	USA	Public	146 (5.6)	4675	32.0	39	141 (96.6)	Kenya (35%, 24.0%)	South Africa (30%, 20.5%)	Uganda (29%, 19.9%)
Fourth	US Department of Health and Human Services	USA	Public	129 (5.0)	4557	35.3	32	114 (88.4)	South Africa (33%, 25.6%)	Nigeria (23%, 17.8%)	Kenya (19%, 14.7%)
Fifth	Fogatry International Center	USA	Public	88 (3.4)	2617	29.7	29	79 (89.8)	Uganda (19%, 21.6%)	Nigeria (17%, 19.3%)	Kenya (13%, 14.8%)
Sixth	Medical Research Council	UK	Public	71 (2.7)	2060	29.0	26	49 (69.0)	South Africa (39%, 54.9%)	Tanzania (21%, 29.6%)	Uganda (11%, 15.5%)
Seventh	Bill and Melinda Gates Foundation	USA	Private	68 (2.6)	2953	43.4	28	43 (63.2)	South Africa (25%, 36.8%)	Uganda (11%, 16.2%)	Rwanda (10%, 14.7%)
Eighth	National Research Foundation	South Africa	Public	53 (2.0)	731	13.8	14	53 (100.0)	South Africa (53%, 100.0%)	Cameroon, Ghana, Kenya, Mozambique, Namibia, Tanzania, Zimbabwe – 1 (1.9%) each	
Ninth	European Commission	Belgium	Public	42 (1.6)	1262	30.0	14	7 (16.7)	South Africa (25%, 59.5%)	Tanzania (8%, 19.0%)	Burkina Faso (6%, 14.3%)
Tenth	Wellcome Trust	UK	Private	41 (1.6)	1222	29.8	15	27 (65.9)	South Africa (13%, 31.7%)	Tanzania (10%, 24.4%)	Uganda (6%, 14.6%)

*Note*: Country 1/2/3, country with the first/second/third highest number of publications supported by the sponsor; *n*, total number of HPV publications in Africa; *N*, total publications per category.

Abbreviations: HQ, headquarters; TP, total publications in Africa; TPAHQCS, total publications with authors from headquarter country of sponsor; TSP, total sponsored publications.

^a^Ranking was based on TPA.

The top 10 funding sponsors of HPV research projects in Africa are depicted in Table 5. Only one African institution—the National Research Foundation (in South Africa)—made the list of these institutions. Most (60%) of these sponsors were based in the United States. Only two (20%) of these sponsors were privately owned. The most prolific sponsor was the National Institutes of Health (TSP = 266; TC = 7860; ACP = 29.5; *h*‐index = 44). The majority (>60%) of the publications generated from these projects had at least one author from the country where the sponsors were based, except for those projects sponsored by the European Commission in which only 16.7% of the total outputs had co‐authors from Belgium. South Africa, Kenya and Nigeria were the most common beneficiaries, as per African country/territory/dependency, of these sponsored projects.

The top 10 journals publishing scholarly outputs on HPV from Africa are depicted in Table S2. All these journals were headquartered in the Global North except two. These two journals were headquartered in Africa and had the lowest CiteScore. The journal with the highest productivity was PLoS One (TP = 116; TC = 1950; ACP = 16.8; *h*‐index = 24; CiteScore (2021) = 5.6).

The top 10 African authors of scholarly publications on HPV are depicted in Table S3. All these authors, except one, were affiliated to the Southern Africa sub‐region. Most were females (80%) and primarily affiliated to South Africa (80%). None of these authors was primarily affiliated with a private institution. Only two (20%) had non‐African foreign affiliations, whereas only three (30%) had a foreign affiliation with an African institution. The most prolific author was Williamson A.L. (TP = 123; TC = 3456; ACP = 28.1; *h*‐index = 35).

**FIGURE 5:**
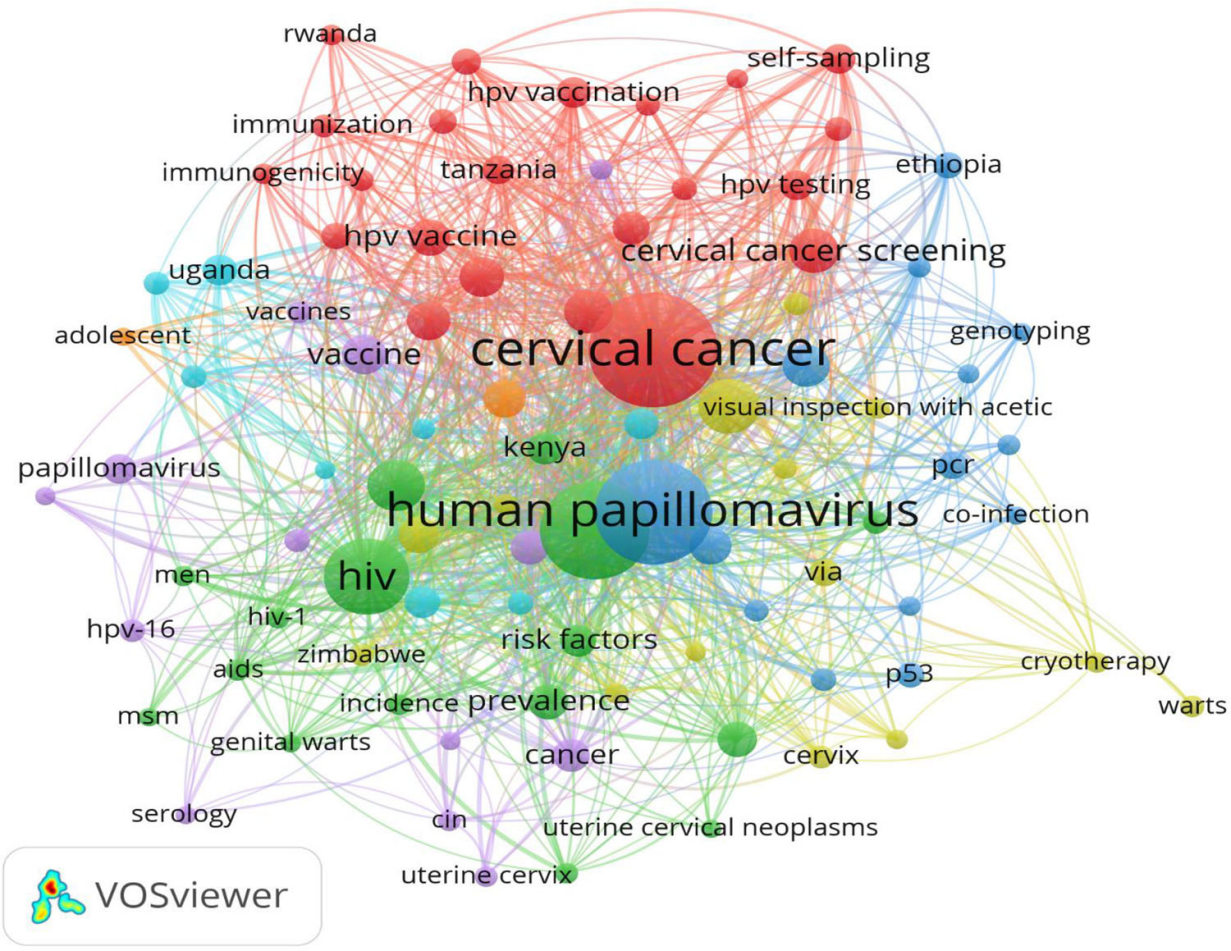
Network visualisation of author keyword co‐occurrence.

Among the top 2000 most‐cited publications on HPV, only 89 keywords occurring in at least 10 publications were analysed through a network visualisation of author keyword co‐occurrence (Figure 5). In the visualisation, the top six author keywords with the highest occurrence rates (OR) and total link strengths (TLS) were ‘cervical cancer’ (OR = 377; TLS = 872), ‘HPV’ (OR = 315; TLS = 625), ‘HPV’ (OR = 271; TLS = 569), ‘HIV’ (OR = 168; TLS = 430), screening (OR = 87; TLS = 267) and ‘Africa’ (OR = 76; TLS = 201). The visualisation has seven clusters. The biggest cluster is depicted in red, and it is made up of 22 author keywords which are predominantly related to cervical cancer prevention (e.g. cervical screening, cervical cancer screening, and HPV test). In comparison, the smallest cluster is depicted in orange and is made up of two author keywords focused on adolescent vaccination (‘adolescent’ and ‘vaccination’).

## DISCUSSION

The findings obtained in this study are very insightful and of public health importance. To start with, the volume of HPV publications in Africa is very low. The African continent comprises 54 countries, 2 dependencies and 2 territories; on average, the mean HPV publication output per African country/dependency/territory is approximately 45 outputs. The HPV publication rate is relatively low in Africa despite carrying the substantial burden of HPV [32, 33, 34]. There have been generally low research capacity and output in Africa [35, 36], not only about HPV. The disproportionately higher burden of HPV has not translated to adequate knowledge production to address the problem. Most significant health problems skew to the Global South, whereas the knowledge solutions flow from the Global North [36]. This has often been a problem since the North controls knowledge production and may be oriented towards the Western global health agenda [37]. This study also revealed that only two indigenous journals made the list of top 10 journals publishing HPV research outputs from Africa.

This study also noted that a few publications (1.2%) were in the Social Sciences. This is also grossly inadequate, even though since the turn of the century, there has been a generally growing social science engagement with health issues—such engagement results from the growing recognition of the complex social nature of health and health care. For instance, qualitative social sciences have explored relational methods to unravel difficult‐to‐measure concepts, such as lived experience, culture, power, inequality and norms [38, 39], among others. As HPV is connected to sexual behaviour, the application of social research is sacrosanct in designing appropriate people‐centred interventions. More so, multidisciplinary efforts will broaden HPV research agenda to have a better understanding in implementing best practices [40]. Based on the above, it will be plausible if research groups or centres of excellence on HPV can be established across multiple research organisations in Africa to boost HPV research productivity in the Social Science discipline as well as other disciplines having low outputs on HPV.

The study also reported inequality in research capacity across the continent. For example the three most prolific African countries in HPV research were South Africa, Nigeria and Egypt. Moreover, all the 10 most prolific researchers were affiliated with public institutions in South Africa, Tanzania and Botswana. Studies have consistently shown these African countries with substantial research output in Africa [41, 42]. Therefore, it is unsurprising that they also top the HPV research output. The study also revealed that the top 10 funding sponsors were public institutions in the United States, United Kingdom, Belgium and South Africa. Research funding is often problematic in African settings. The study revealed inequality in HPV funding, with researchers and institutions affiliated to South Africa, Nigeria, Kenya and Uganda constituting the predominant beneficiaries.

In most cases, there is an international priority to conduct research in Africa and provide funding. Still, most developing countries often have low local funding for research and development (R&D) [43]. Most African nations are yet to meet the target of one per cent of GDP for R&D [43]. Although for the main time, international donors can continue to support HPV research, African nations need to increase local capacities and ‘sustain world‐class research hubs that will be conducive to address Africa's intractable health challenges’ [35], including HPV. Health research, funding and research capacity must be improved to tackle unmet health needs in Africa [43].

Pertinently, this study also identified that the top HPV researchers in Africa were women. This observation may be because women are the population group that are most adversely affected by HPV infection [10, 11, 12, 13, 14, 15, 16]; hence, this might have kindled the interests of female researchers to focus on the area. Furthermore, this research interest area, perhaps, is one of the few areas dominated by women [44, 45, 46]; this suggests that HPV research is a niche where women researchers are thriving. Unfortunately, research project funding has so much prioritised projects led by men [44]. This may suggest why there has been a relatively low funding rate for HPV research in Africa. This underscores the need for increased funding for women‐led HPV research projects in Africa.

As evidenced by the network visualisation of keyword co‐occurrence, the hottest HPV‐related research area in Africa was cervical cancer. There were low outputs on HPV‐associated head‐and‐neck cancer, HPV vaccination and other pertinent areas on HPV. The low productivity rate on the aforementioned focus areas makes it difficult for Africa to achieve a holistic and formidable front that can achieve the local, national, regional and global goals of HPV eradication [47]. Therefore, there is a need to rejuvenate research interests on this focus area across Africa, and this can be achieved through the provision of research capacity development in these areas through research funding, research fellowship programmes, mentorships and networking [48].

However, this study has its limitation. This bibliometric review relied on a single database; hence, those publications not indexed in SCOPUS were excluded in the review. Multiple databases would have been included in this review; however, such was not done because the use of multiple databases in bibliometric reviews significantly limits the opportunity for a robust and in‐depth data analysis such as network visualisations [26, 27, 28, 29, 30, 31]. Hence, the findings presented in this study should be treated with caution.

## CONCLUSION

Scholarly HPV research productivity in Africa is very low and worsened by multiple inequality gaps, including geographical distribution of the outputs and funding. Despite the high burden of HPV in Africa, there are no commensurate research efforts to tackle the problem. Like other health conditions, the Global North controls the production and flow of knowledge concerning HPV. There are a limited number of journals dedicated to HPV research in Africa. The inequality in HPV research is also evident across the African continent, with a high concentration of research outs in three countries: South Africa, Nigeria and Egypt. The study observed gaps in multidisciplinary research, mainly due to low outputs from the health social sciences. Meanwhile, the application of social research is essential to control risk behaviour and for implementation research. The findings signify an urgent need for equitably strengthening HPV research capacity in Africa.

## AUTHOR CONTRIBUTIONS

*Conceptualisation; data curation; formal analysis; funding acquisition; investigation; methodology; project administration; resources; software; supervision; validation; visualisation; writing – original draft; writing – review and editing*: Kehinde Kazeem Kanmodi. *Data curation; formal analysis; resources*: Eyinade Adeduntan Egbedina. *Writing – original draft; writing – review and editing*: Jimoh Amzat. *Resources*: Kafayat Aminu. *Resources*: Lawrence Achilles Nnyanzi.

## CONFLICT OF INTEREST STATEMENT

The authors declare no conflicts of interest.

## Supporting information

Supporting Information

## Data Availability

Data sharing is not applicable to this article as no new data were created or analysed in this study.
