# Supplementary material for: The state of human papillomavirus research in Africa
Source: Public Health Chall. 2023 Mar 3;2(1):e72. doi: 10.1002/puh2.72 (PMC12039679; doi:10.1002/puh2.72)
Supplement: Supplementary file 1 — Supporting Information [file PUH2-2-e72-s001.docx]

**Box 1. Search string used for scooping out HPV literature from Africa**

| ( ( TITLE-ABS-KEY ( hpv )  OR  TITLE-ABS-KEY ( human  AND papillomavir* )  OR  TITLE-ABS-KEY ( human  AND papilloma  AND vir* ) ) )  AND  ( ( ( AFFILCOUNTRY ( angola )  OR  AFFILCOUNTRY ( benin )  OR  AFFILCOUNTRY ( botswana )  OR  AFFILCOUNTRY ( burkina  AND  faso )  OR  AFFILCOUNTRY ( burundi )  OR  AFFILCOUNTRY ( cameroon )  OR  AFFILCOUNTRY ( cabo  AND  verde )  OR  AFFILCOUNTRY ( cape  AND  verde )  OR  AFFILCOUNTRY ( central  AND  african  AND  republic )  OR  AFFILCOUNTRY ( chad )  OR  AFFILCOUNTRY ( comoros )  OR  AFFILCOUNTRY ( congo )  OR  AFFILCOUNTRY ( ivory  AND  coast )  OR  AFFILCOUNTRY ( democratic  AND  republic  AND  of  AND  congo )  OR  AFFILCOUNTRY ( djibouti )  OR  AFFILCOUNTRY ( equatorial  AND  guinea )  OR  AFFILCOUNTRY ( eritrea )  OR  AFFILCOUNTRY ( ethiopia )  OR  AFFILCOUNTRY ( gabon )  OR  AFFILCOUNTRY ( gambia )  OR  AFFILCOUNTRY ( ghana )  OR  AFFILCOUNTRY ( guinea )  OR  AFFILCOUNTRY ( guinea-bissau )  OR  AFFILCOUNTRY ( kenya )  OR  AFFILCOUNTRY ( lesotho )  OR  AFFILCOUNTRY ( liberia )  OR  AFFILCOUNTRY ( madagascar )  OR  AFFILCOUNTRY ( malawi )  OR  AFFILCOUNTRY ( mali )  OR  AFFILCOUNTRY ( mauritania )  OR  AFFILCOUNTRY ( mauritius )  OR  AFFILCOUNTRY ( mayotte )  OR  AFFILCOUNTRY ( mozambique )  OR  AFFILCOUNTRY ( namibia )  OR  AFFILCOUNTRY ( niger )  OR  AFFILCOUNTRY ( nigeria )  OR  AFFILCOUNTRY ( reunion )  OR  AFFILCOUNTRY ( rwanda )  OR  AFFILCOUNTRY ( saint  AND  helena )  OR  AFFILCOUNTRY ( sao  AND  tome  AND  principe )  OR  AFFILCOUNTRY ( senegal )  OR  AFFILCOUNTRY ( seychelles )  OR  AFFILCOUNTRY ( sierra  AND  leone )  OR  AFFILCOUNTRY ( somalia )  OR  AFFILCOUNTRY ( south  AND  africa )  OR  AFFILCOUNTRY ( south  AND  sudan ) ) )  OR  ( ( AFFILCOUNTRY ( eswatini )  OR  AFFILCOUNTRY ( togo )  OR  AFFILCOUNTRY ( uganda )  OR  AFFILCOUNTRY ( zambia )  OR  AFFILCOUNTRY ( zimbabwe )  OR  AFFILCOUNTRY ( egypt )  OR  AFFILCOUNTRY ( libya )  OR  AFFILCOUNTRY ( algeria )  OR  AFFILCOUNTRY ( tunisia )  OR  AFFILCOUNTRY ( morocco )  OR  AFFILCOUNTRY ( western  AND  sahara )  OR  AFFILCOUNTRY ( sudan )  OR  AFFILCOUNTRY ( tanzania ) ) ) ) |
| --- |

**Table S1. Top-three most productive African institutions**

| **Institution (n=2587)** | **Rank*** | **Country** | **Ownership** | **Total Publications (%)** | **Total Citations** | **Average Citation per Publication** | **h-Index** |
| --- | --- | --- | --- | --- | --- | --- | --- |
| **Top-three African Institutions** | | | | | | | |
| University of Cape Town | 1^st^ | South Africa | Public | 335 (12.9) | 11,355 | 33.9 | 55 |
| National Health Laboratory Services | 2^nd^ | South Africa | Public | 205 (7.9) | 3,867 | 18.9 | 35 |
| University of Witwatersrand | 3^rd^ | South Africa | Public | 166 (6.4) | 3,590 | 21.6 | 31 |
| **Top-three Institutions in North Africa** | | | | | | | |
| Cairo University | 1^st^ | Egypt | Public | 45 (1.7) | 587 | 13.0 | 15 |
| Zagazig University | 2^nd^ | Egypt | Public | 35 (1.4) | 285 | 8.1 | 7 |
| Alexandria University | 3^rd^ | Egypt | Public | 31 (1.2) | 492 | 15.9 | 12 |
| **Top-three Institutions in West Africa** | | | | | | | |
| University of Ibadan | 1^st^ | Nigeria | Public | 51 (2.0) | 2,398 | 47.0 | 16 |
| University College Hospital | 2^nd^ | Nigeria | Public | 27 (1.0) | 400 | 14.8 | 10 |
| Institute of Human Virology – Nigeria | 3^rd^ | Nigeria | Public | 26 (1.0) | 1,201 | 46.2 | 13 |
| **Top-three Institutions in Central Africa** | | | | | | | |
| University of Dschang | 1^st^ | Cameroon | Public | 21 (0.8) | 100 | 4.8 | 5 |
| University of Rwanda | 2^nd^ | Rwanda | Public | 18 (0.7) | 272 | 15.1 | 10 |
| University of Yaounde I | 3^rd^ | Cameroon | Public | 17 (0.7) | 182 | 10.7 | 8 |
| **Top-three Institutions in East Africa** | | | | | | | |
| Makerere University | 1^st^ | Uganda | Public | 95 (3.7) | 4,747 | 50.0 | 31 |
| Kenya Medical Research Institute | 2^nd^ | Kenya | Public | 69 (2.7) | 1,087 | 15.8 | 19 |
| University of Nairobi | 3^rd^ | Kenya | Public | 62 (2.4) | 1,455 | 23.5 | 22 |
| **Top-three Institutions in Southern Africa** | | | | | | | |
| University of Cape Town | 1^st^ | South Africa | Public | 335 (12.9) | 11,355 | 33.9 | 55 |
| National Health Laboratory Services | 2^nd^ | South Africa | Public | 205 (7.9) | 3,867 | 18.9 | 35 |
| University of Witwatersrand | 3^rd^ | South Africa | Public | 166 (6.4) | 3,590 | 21.6 | 31 |
| n – Total number of HPV publications in Africa; *Ranking was based on TP  NB: Data was based on institutional affiliations of the analysed HPV publications | | | | | | | |

**Table S2. The top ten journals publishing scholarly outputs on HPV from Africa**

| **Journal (n=2549)** | **Publisher (HQ Country)** | **CiteScore (2021)** | **Total Publications (%)** | **Total Citations** | **Average Citation per Publication** | **h-Index** |
| --- | --- | --- | --- | --- | --- | --- |
| PLoS One | Public Library of Science (USA) | 5.6 | 116 (4.6) | 1950 | 16.8 | 24 |
| Infectious Agents and Cancer | Springer Nature (UK) | 4.5 | 87 (3.4) | 1103 | 12.7 | 19 |
| Vaccine | Elsevier (Netherlands) | 6.7 | 76 (3.0) | 3016 | 39.7 | 29 |
| South African Medical Journal | South African Medical Association (South Africa) | 2.4 | 58 (2.3) | 546 | 9.4 | 14 |
| International Journal of Cancer | Wiley-Blackwell (USA) | 12.4 | 54 (2.1) | 3899 | 72.2 | 33 |
| International Journal of Gynecology and Obstetrics | Wiley-Blackwell (USA) | 5.7 | 53 (2.1) | 958 | 18.1 | 18 |
| Pan African Medical Journal | Pan African Medical Journal (Nigeria) | 1.0 | 52 (2.0) | 264 | 5.1 | 8 |
| Journal of Medical Virology | Wiley-Blackwell (USA) | 18.8 | 44 (1.7) | 901 | 20.5 | 19 |
| Journal of Infectious Diseases | Oxford University Press (UK) | 10.3 | 34 (1.3) | 1657 | 48.8 | 24 |
| Sexually Transmitted Diseases | Wolters Kluwer Health (Netherlands) | 3.5 | 32 (1.3) | 608 | 19.0 | 14 |
| n – Total number of HPV journal publications in Africa; *Ranking was based on TP | | | | | | |

**Table S3. The top ten African authors of scholarly publications on HPV**

| **Rank*** | **Author (n=2587)** | **Gender** | **Primary Affiliation**** | **Ownership** | **Country** | **AS** | **HFNA** | **HFAA** | **TP (%)** | **YFP** | **YMRP** | **TC** | **ACP** | **h-index** |
| --- | --- | --- | --- | --- | --- | --- | --- | --- | --- | --- | --- | --- | --- | --- |
| 1^st^ | Williamson A.L. | Female | University of Cape Town (1989 – 2022) | Public | South Africa | Southern Africa | Yes | Yes | 123 (4.8) | 1991 | 2022 | 3,456 | 28.1 | 35 |
| 2^nd^ | Denny L.A. | Female | University of Cape Town (1994 – 2022) | Public | South Africa | Southern Africa | Yes | No | 81 (3.1) | 2000 | 2022 | 4,847 | 59.8 | 33 |
| 3^rd^ | Rybicki E.P. | Male | University of Cape Town (1981 – 2022) | Public | South Africa | Southern Africa | Yes | No | 40 (1.5) | 1991 | 2020 | 1,690 | 42.3 | 24 |
| 4^th^ | Watson-Jones D. | Female | National Institute for Medical Research (2000 – 2022) | Public | Tanzania | East Africa | Yes | Yes | 33 (1.3) | 2012 | 2022 | 998 | 30.2 | 18 |
| 5^th^ | Allan B.R. | Male | University of Cape Town (2000 – 2017) | Public | South Africa | Southern Africa | No | No | 27 (1.0) | 2000 | 2017 | 924 | 34.2 | 18 |
| 5^th^ | Hitzeroth I | Female | University of Cape Town (1993 – 2022) | Public | South Africa | Southern Africa | Yes | No | 27 (1.0) | 2006 | 2022 | 846 | 31.3 | 15 |
| 5^th^ | Mbulawa Z.Z.A. | Female | University of Cape Town & National Health Laboratory Services (2008 – 2022) | Public | South Africa | Southern Africa | No | No | 27 (1.0) | 2008 | 2022 | 422 | 15.6 | 12 |
| 8^th^ | Michelow P.M. | Female | University of Witwatersrand & National Health Laboratory Services (1995 – 2022) | Public | South Africa | Southern Africa | Yes | No | 25 (1.0) | 2009 | 2021 | 399 | 16.0 | 10 |
| 9^th^ | Ramogola-Masire D. | Female | University of Botswana (2011 – 2022) | Public | Botswana | Southern Africa | Yes | Yes | 23 (0.9) | 2010 | 2022 | 264 | 11.5 | 9 |
| 10^th^ | Delany-Moretlwe S. | Female | University of Witwatersrand (2004 – 2022) | Public | South Africa | Southern Africa | Yes | No | 22 (0.9) | 2013 | 2022 | 236 | 10.7 | 9 |
| **The institution having the longest duration of which the author has published with—this information was obtained from author’s profile page on SCOPUS; HFNA – History of Foreign non-African Affiliation; HFAA – History of Foreign Affiliation within Africa; TP – Total publication; YFP – Year of first publication; YLP – Year of most recent publication; TC – Total citations; ACP – Average citation per paper; AS – African Subregion; *Ranking was based on TP; n – Total number of HPV publications in Africa | | | | | | | | | | | | | | |
